# Safety Profile and Immunologic Responses of a Novel Vaccine Against *Shigella sonnei* Administered Intramuscularly, Intradermally and Intranasally: Results From Two Parallel Randomized Phase 1 Clinical Studies in Healthy Adult Volunteers in Europe

**DOI:** 10.1016/j.ebiom.2017.07.013

**Published:** 2017-07-15

**Authors:** Odile Launay, David J.M. Lewis, Alessandra Anemona, Pierre Loulergue, Jo Leahy, Antonella Silvia Sciré, Anaïs Maugard, Elisa Marchetti, Stefano Zancan, Zhiming Huo, Simona Rondini, Rachid Marhaba, Oretta Finco, Laura B. Martin, Jochen Auerbach, Daniel Cohen, Allan Saul, Christiane Gerke, Audino Podda

**Affiliations:** a Université Paris Descartes, Sorbonne Paris cité, Paris, France; b University of Surrey, Guildford, United Kingdom; c GSK Vaccines Institute for Global Health, Siena, Italy; d GSK Vaccines, Siena, Italy; e GSK Vaccines, Marburg, Germany; f Tel Aviv University School of Public Health, Tel Aviv, Israel; g Inserm CIC 1417, Paris, France; h Assistance Publique Hôpitaux de Paris, CIC Cochin-Pasteur, Paris, France

**Keywords:** *Shigella sonnei*, Vaccine, Clinical study, GMMA

## Abstract

**Background:**

Approximately 164,000 deaths yearly are due to shigellosis, primarily in developing countries. Thus, a safe and affordable *Shigella* vaccine is an important public health priority. The GSK Vaccines Institute for Global Health (GVGH) developed a candidate *Shigella sonnei* vaccine (1790GAHB) using the Generalized Modules for Membrane Antigens (GMMA) technology. The paper reports results of 1790GAHB Phase 1 studies in healthy European adults.

**Methods:**

To evaluate the safety and immunogenicity profiles of 1790GAHB, we performed two parallel, phase 1, observer-blind, randomized, placebo-controlled, dose escalation studies in France (“study 1”) and the United Kingdom (“study 2”) between February 2014 and April 2015 (ClinicalTrials.gov, number NCT02017899 and NCT02034500, respectively) in 18–45 years old subjects (50 in study 1, 52 in study 2). Increasing doses of Alhydrogel adsorbed 1790, expressed by both O Antigen (OAg) and protein quantity, or placebo were given either by intramuscular route (0.059/1, 0.29/5, 1.5/25, 2.9/50, 5.9/100 μg of OAg/μg of protein; study 1) or by intradermal (ID), intranasal (IN) or intramuscular (IM) route of immunization (0.0059/0.1, 0.059/1, 0.59/10 μg ID, 0.29/5, 1.2/20, 4.8/80 μg IN and 0.29/5 μg IM, respectively; study 2). In absence of serologic correlates of protection for *Shigella sonnei*, vaccine induced immunogenicity was compared to anti-LPS antibody in a population naturally exposed to *S. sonnei*.

**Findings:**

Vaccines were well tolerated in both studies and no death or vaccine related serious adverse events were reported. In study 1, doses ≥ 1.5/25 μg elicited serum IgG median antibody greater than median level in convalescent subjects after the first dose. No vaccine group in study 2 achieved median antibody greater than the median convalescent antibody.

**Interpretation:**

Intramuscularly administered *Shigella sonnei* GMMA vaccine is well tolerated, up to and including 5.9/100 μg and induces antibody to the OAg of at least the same magnitude of those observed following natural exposure to the pathogen. Vaccine administered by ID or IN, although well tolerated, is poorly immunogenic at the doses delivered. The data support the use of the GMMA technology for the development of intramuscular multivalent *Shigella* vaccines.

## Highlights

• GVGH GMMA vaccine against *S. sonnei* was well tolerated by IM, IN and ID routes of immunization in young European adults.
• Doses ≥ 1.5/25 μg elicited serum IgG median antibody greater than median level in convalescent subjects after the first dose.
• Clinical data support the use of the GMMA technology for the development of intramuscular *Shigella* multivalent vaccines.

Shigellosis is an important cause of diarrhoea especially in children of developing countries. No vaccine is available. Based on the 2015 Global Burden of Disease, *Shigella* caused approximately 164,000 deaths due to diarrhoea in 2015, supporting the public health relevance of the disease and the need for a vaccine. The GMMA (Generalized Modules for Membrane Antigens) technology was used for the development of a vaccine against *Shigella sonnei* which, tested in these trials for the first time, was shown to be well tolerated in young adults and induced specific antibody titres at least as high as after natural infection.

## 1 Introduction

*Shigella* infections are endemic throughout the world, but the main disease burden is in developing countries, especially in children younger than 5 years of age (Kotloff et al., 1999). The recently published Global Burden of Disease Study 2015 estimates that 12.5% (i.e., 164,410) of the 1.3 million deaths due to diarrheal diseases were caused by *Shigella* (GBD 2015 Mortality and Causes of Death Collaborators, 2016). In that study, 98.5% of *Shigella* deaths occurred in low and middle income countries and 33% in children younger than 5 years old. These data agree with data from more geographically limited studies: the estimated incidence of disease in Asia alone is approximately 125 million cases per year (Bardhan et al., 2010), with approximately 122,000 annual deaths (Lozano et al., 2012).

These estimates are supplemented with incidence data from specific sites of the Global Enteric Multicentre Study (GEMS) in sub-Saharan Africa and South Asia (Kotloff et al., 2013) that found that shigellosis is one of the top causes of moderate and severe diarrhoea (MSD) in under 5 year olds. *Shigella* was the fourth, second and first cause of medium or severe diarrhoea (MSD), in children aged 0–11 months, 12–23 months and 24–59 months, respectively (Kotloff et al., 2013). Of 1124 *Shigella* cases in the GEMS study 23.9% were caused by *S. sonnei* (single serotype), 5.5% by *S. boydii* (multiple serotypes), 4.9% by *S. dysenteriae* (multiple serotypes, but no cases of *S. dysenteriae I*) and 65.7% by *S. flexneri* (mostly by serotypes 1b, 2a, 3a, and 6) (Livio et al., 2014). A subsequent re-analysis of GEMS data with more sensitive molecular diagnostic techniques (Lindsay et al., 2013, Liu et al., 2014) showed an even higher incidence of *Shigella* as a cause of MSD. These data from developing countries and evidence of increasing levels of antibiotic resistance (Gu et al., 2012, Klontz and Singh, 2015) support the need for a broadly protective vaccine for efficient prevention of the disease (Livio et al., 2014).

*S. sonnei* alone causes significant disease in industrialized countries and travellers, and in these populations is responsible for 60–70% of all cases of shigellosis (Ekdahl and Andersson, 2005). Antibiotic resistant strains of *S. sonnei* also cause disease and local outbreaks in people who have not travelled to endemic countries (Bowen et al., 2015). Thus, a *S. sonnei* monovalent vaccine may also have public health value.

No vaccine is widely available to prevent shigellosis (Mani et al., 2016). So far, vaccine candidates based on O Antigen (OAg) conjugates and live attenuated strains have shown protection against homologous strains in Phase 3 clinical studies (Cohen et al., 1997, Levine et al., 2007, Kaminski and Oaks, 2009, Camacho et al., 2013, Passwell et al., 2010). Vaccines using inactivated bacteria and vaccine candidates based on sub-cellular components are currently under development. Most of these also contain OAg (Levine et al., 2007, Kaminski and Oaks, 2009, Camacho et al., 2013).

The central role of the serotype specific OAg repeating unit of *Shigella* LPS in clinical protection has been demonstrated in various settings. More specifically, i) subjects naturally exposed to *Shigella* develop a serotype-specific immunity, but remain susceptible to disease caused by heterologous serotypes (Ferreccio et al., 1991); ii) subjects previously infected/challenged with either *S. sonnei* or *S. flexneri* develop protection only against the challenge strain as shown with re-challenge experiments (Kotloff et al., 1995, Herrington et al., 1990); iii) subjects lacking homologous anti-LPS antibodies have a significantly higher risk of contracting shigellosis of the same serovar type (Cohen et al., 1991); iv) subjects with a high level of anti-LPS serum antibodies show significantly reduced disease severity (Wahid et al., 2013); and v) subjects vaccinated IM with a conjugate vaccine containing *Shigella sonnei* O polysaccharide bound to *Pseudomonas aeruginosa* recombinant exoprotein A develop a serotype specific 71–74% efficacy in older children and adults and show a significant association between anti-*S. sonnei* LPS serum antibody and clinical protection (Cohen et al., 1997, Passwell et al., 2010). These findings demonstrate that natural immunity against *Shigella* is primarily serotype-specific; the OAg of the *Shigella* LPS is the dominant protective antigen and anti-*S. sonnei* LPS antibody is a strong marker of acquired immunity.

The vaccine development approach pursued by the GSK Vaccines Institute for Global Health (GVGH) is based on Generalized Modules for Membrane Antigens (GMMA). GMMA are approximately 50 nm outer membrane blebs derived from bacteria genetically modified to enhance shedding and to reduce reactogenicity of the integral lypopolisaccharide. Unlike detergent extracted OMV, these contain all of the components of the bacterial outer membrane and enclose bacterial periplasm (Gerke et al., 2015, Maggiore et al., 2016). GVGH has developed a monovalent *Shigella sonnei* vaccine with OAg (1790GAHB) as the first step in the development of a broadly protective, multivalent vaccine including GMMA with OAg from different *Shigella* serotypes. In this paper, we report the results from two parallel Phase 1 clinical studies performed in healthy adult volunteers in Europe with 1790GAHB vaccine. In absence of an accepted serologic threshold of protection for *Shigella sonnei*, we compare the vaccine-induced anti-*S. sonnei* LPS antibody with the levels and distribution in Israeli convalescent subjects after natural infection (Cohen et al., 1989).

## 2 Methods

### 2.1 Study Design and Participants

Between February 2014, and April 2015, we performed two parallel, phase 1, observer-blind, randomized, placebo-controlled, dose escalation studies: study 1 (H03_01TP) at one site in France and study 2 (H03_02TP) at one site in the United Kingdom. The dose escalation approach reflected the caution needed in Phase 1 studies for the safety evaluation of a novel vaccine. The two studies are registered with ClinicalTrials.gov, numbers NCT02017899 and NCT02034500. We enrolled healthy adults (aged 18–45 years old), as established by medical history, physical examination, screening for relevant haematology, blood chemistry and urinalysis tests, investigator judgment, and compliance with protocol inclusion and exclusion criteria. A qualitative agglutination test was performed by investigators at baseline as part of the screening to exclude subjects with high antibody titres against *S. sonnei*. Eligible women had negative results on pregnancy tests, before each vaccination, and agreed using an acceptable birth control measure during study participation. Relevant approvals were obtained before study start from respective institutional ethics review committees and national regulatory authorities. An independent data and safety monitoring board (DSMB) was appointed to oversee safety of study participants. During studies, DSMB met after safety data from each cohort became available, to assess the safety profile of study vaccine and advise on progression to subsequent cohorts. Written informed consent was obtained before enrolment. Studies were designed and conducted in accordance with Good Clinical Practice and current version of Declaration of Helsinki.

### 2.2 Vaccine

Details of the production of the vaccine, and pre-clinical studies have been published (Gerke et al., 2015). Briefly, the test vaccine was a preservative-free, liquid formulation of *S. sonnei* 1790-GMMA containing 11.9 μg/mL of OAg and 200 μg/mL protein adsorbed to 0.7 mg Al^3 +^/mL Aluminium hydroxide (Alhydrogel 2%, Brenntag Biosector, Denmark) in Tris-buffered saline, pH 7.4 (TBS) in single dose vials containing 0.7 mL of vaccine. The vaccine was used at different antigen doses (that were obtained by bed-side mixing using placebo as diluent). For a more precise information about vaccine composition, each dose is defined by both OAg and protein quantity: a dose containing 5.9 μg of OAg and 100 μg protein is specified as 5.9/100 μg. The clinical lot (SH-13-002) was the same for both studies. In the absence of a licensed *S. sonnei* vaccine, the control agent was a placebo of Alhydrogel (0.7 mg Al^3 +^/mL) in TBS, as single dose vials containing 0.7 mL of injectable solution. The placebo administration volume depended on the route of administration and was the same as the vaccine.

### 2.3 Randomization and Masking

In study H03_01TP, a total of 50 eligible subjects were assigned to 1 of 5 sequential cohorts of 10 subjects. Within each cohort, subjects were randomized with a 4:1 ratio to receive 3 vaccinations IM, 4 weeks apart, of either 1790GAHB vaccine (8 subjects) or placebo (2 subjects). Cohorts were vaccinated sequentially with a dose escalating approach with 0.059/1, 0.29/5, 1.5/25, 2.9/50 or 5.9/100 μg in an injected volume of 0.5 mL.

**Fig. 1:**
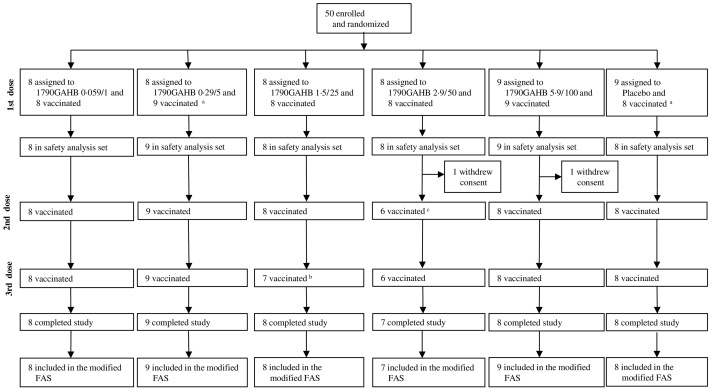
Study 1 - H03_01TP trial profile. ^a^One subject was randomized to receive placebo but he/she received 3 vaccinations of 0.29/5 μg *S. sonnei* vaccine. ^b^One subject received vaccine forbidden by the protocol and didn't receive third vaccination. ^c^One subject developed neutropenia after the first vaccination and he/she didn't receive the following vaccinations. FAS = full analysis set. The analysis of immunogenicity was based on modified FAS defined as: all randomized subjects who received at least one study vaccination and provided immunogenicity data at relevant time points. Subjects who received wrong vaccine at all vaccinations were analysed in the vaccine the subject actually received and blood samples collected after a vaccination visit but vaccine was not administered, were excluded from the analysis.

**Fig. 2:**
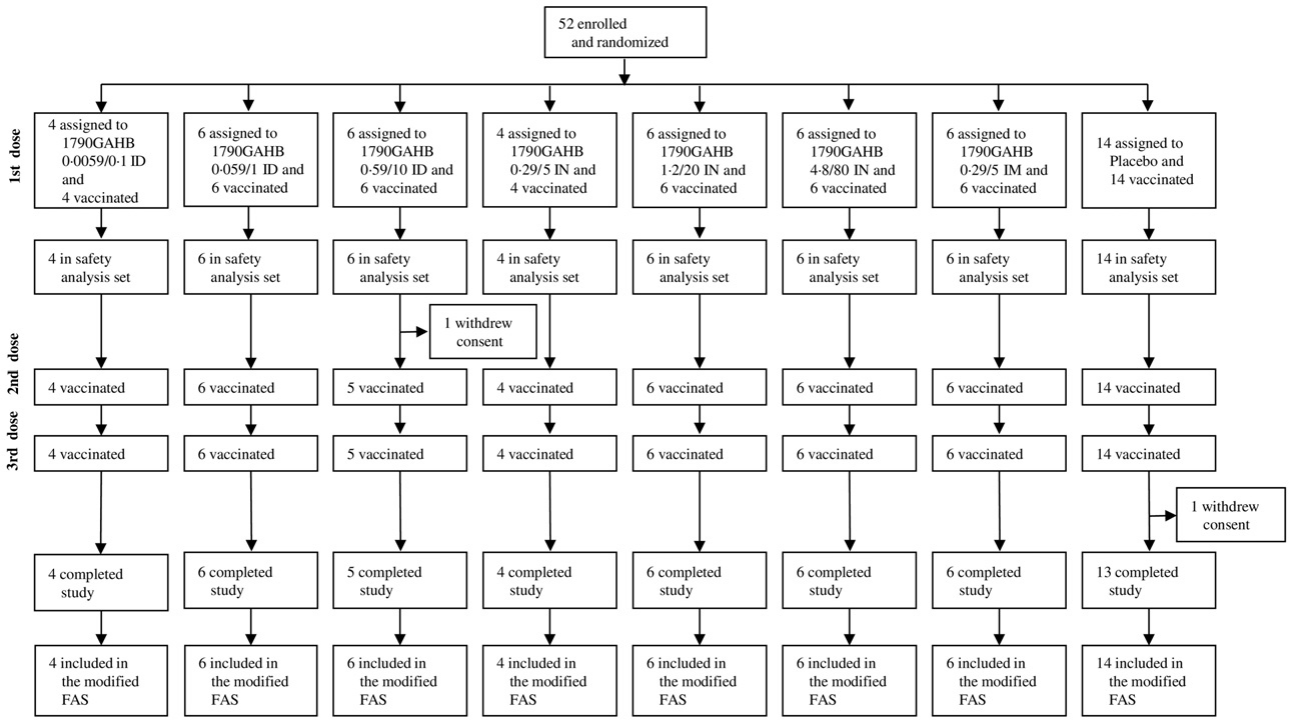
Study 2 - H03_02TP trial profile. FAS = full analysis set. The analysis of immunogenicity was based on modified FAS defined as: all randomized subjects who received at least one study vaccination and provided immunogenicity data at relevant time points. Subjects who received wrong vaccine at all vaccinations were analysed in the vaccine the subject actually received and blood samples collected after a vaccination visit but vaccine was not administered, were excluded from the analysis.

In study H03_02TP, a total of 52 eligible subjects were assigned to 3 cohorts to receive with a 2:1 ratio (cohort 1) or a 3:1 ratio (cohorts 2 and 3) either 1790GAHB vaccine or placebo in 3 vaccinations, 4 weeks apart. Cohorts were vaccinated sequentially with a dose escalating approach with individuals within each cohort receiving 0.0059/0.1, 0.059/1, and 0.59/10 μg ID in 0.05 mL or 0.29/5, 1.2/20, and 4.8/80 μg IN in a total volume of 0.4 mL administered as a 0.2 mL aliquot into each nostril using a mucosal atomization device (Wolfe Tory Medical, Inc., Utah, USA). In addition, 8 subjects of the third cohort were randomized with a 3:1 ratio to receive three IM vaccinations, four weeks apart, of 0.29/5 μg of 1790GAHB or placebo. Fig. 1, Fig. 2 show the flow chart of the two studies.

With the exception of designated study site personnel responsible for vaccine preparation (not involved in data collection or assessment), study investigators, participants, those assessing outcomes, and data analysts were blinded to treatment allocation.

## 3 Procedures

Subjects were observed at the clinic for 4 h in study 1 and 2 h in study 2 after each vaccination and then at 7 days following the first vaccination. Safety assessment included collection of solicited adverse events (AEs), unsolicited AEs, and medication/vaccinations during 7 days following each vaccination; additionally, all serious adverse events (SAEs), all medications given to treat SAEs, all new onset of chronic disease (NOCDs), all AEs leading to vaccine/study withdrawal, and all adverse event of special interest (AESI) were collected for the entire study duration. In study 1, ear temperatures were measured by clinical staff immediately prior to vaccination, then at 30, 120 and 240 minute post-vaccination, then oral temperature at 6 h and daily to day 7 by the subject and recorded on their diary cards. In study 2, ear temperature was measured prior to vaccination then at 30 min and 120 h by clinical staff, then as for study 1, oral temperature was measured by subjects at 6 h then daily and recorded on their dairy cards. All temperature readings were used to determine a fever episode (defined as a temperature > 38 °C) and the 0 to 240 minute temperature recordings in study 1 were analysed for any evidence of a pyrogenic response to the intramuscular vaccine.

Clinically significant deviations in haematological, biochemical and urinalysis tests were evaluated during the screening, at 7 days after first vaccination and 28 days after second and third vaccination. Subsequent to two cases of transient and clinically asymptomatic grade 3 neutropenia (Muturi-Kioi et al., 2016), a complete blood count (CBC) was also performed 7 days after second and third vaccinations and 168 days after third vaccination.

For immunological studies, blood draws and stool samples were obtained before each study vaccination and 28 days after the first, second and third vaccination, and 168 days after the third vaccination (Study day 225). Faecal sIgA, have been analysed, in study subjects from cohort 3 of study 2 at Surrey University (extractions) and at GVGH (ELISA). Exploratory immunological outcomes, only for cohort 3 of study 2, included assessment of memory B cells (MBC), specific for the OAg and its GMMA carrier, and of antibody secreting cells (plasmablasts) on PBMCs obtained at baseline and at 28 days after the third immunization (MBC) and at 7 days after the first immunization (plasmablasts). Assays were performed at GSK Vaccines, Siena, Italy, by ELISpot, following standard procedures (Slifka and Ahmed, 1996). Serum anti-*S. sonnei* LPS IgG was evaluated by ELISA at the GSK Vaccines, Clinical Laboratory Sciences Department (Marburg, Germany), as previously described (Gerke et al., 2015).

## 4 Statistical Analysis

These phase 1 safety and immunogenicity trials were aimed to descriptively evaluate the safety and immunogenicity profiles of the study vaccines without testing any specific hypotheses. For ELISA testing, the minimum measurable antibody level was determined in each assay and varied from 3.08 to 4.06 ELISA units. For calculating geometric mean antibody levels, subjects below this level were assigned an antibody level of half the minimum measurable ELISA units. Subjects defined as positive for pre-existing antibody had a baseline anti-LPS IgG greater than the minimum measurable ELISA units. We defined “seroresponders” as individuals with a change of antibody concentration of at least 25 ELISA units or a 50% increase in antibody over baseline levels, whichever was greater. As a guide to the relevance of the magnitude of the IgG responses, the median anti-*S. sonnei* LPS serum IgG concentration was compared to the median level in convalescent patient sera from 87 subjects previously infected with *S. sonnei*, as reported by Cohen et al. (1989) To do this, we calibrated the GVGH standard human anti-*S. sonnei* LPS antisera in the Cohen ELISA assay and found that 121 ELISA units in the GVGH assay correspond to the median 1:800 titer reported in the convalescent sera. As defined by the protocol, as a secondary indicator, we also tabulated the number and percentages of subjects with post-immunization ELISA units ≥ 121 EU/mL (i.e., high seroresponse) and used 2-sided 95% Clopper-Pearson to calculate the CIs for the percentages. However, unexpectedly, there were 5 of 50 and 2 of 52 subjects, in study 1 and study 2 respectively, who had a “high seroresponse” prior to vaccination. Thus in this paper we additionally define a “high seroresponder” as a volunteer with an increase of at least 121 ELISA units over baseline. Finally, some post hoc analyses have been conducted for a better interpretation of the safety and immunogenicity results and are reported in the Results.

## 5 Results

### 5.1 Recruitment

Reasons for screening failures included: previous illness/investigator judgment, non-availability for study duration, HLA-B27 positivity, receipt of other vaccines/previous participation in other studies, and serum positivity by *Shigella* agglutination assay. Only two subjects (1 in each study) were excluded for the latter reason. The presence of detectable antibodies at baseline in approximately 50% of the subjects, as shown in the ELISA analyses, suggests that this agglutination assay has a low sensitivity and should not be used in future studies. Of the 102 subjects enrolled and vaccinated in the two studies (50 in study 1; 52 in study 2), 48 in study 1 and 50 in study 2 completed the study. Two subjects in each study withdrew their consent before the end of the study. All subjects were included in safety analyses and all, but one (in study 1), were included in the modified Full Analysis Set (FAS; Supplementary Fig. 1, Fig. 2). As shown in Tables 1 and 2, for study 1 and 2 respectively, the baseline characteristics of subjects were similar across vaccine and placebo groups.

**Table 1:** Baseline characteristics in study 1.

	IM					
	0.059/1	0.29/5	1.5/25	2.9/50	5.9/100	Placebo
n	8	9	8	7	9	8
Age (years)	32.1 (6.7)	29.8 (7.0)	26.8 (5.7)	33.4 (8.1)	26.9 (4.2)	33.1 (8.2)
Weight (kg)	68.20 (13.95)	69.00 (8.40)	68.55 (10.87)	58.93 (6.94)	63.77 (10.51)	71.89 (10.41)
Height (cm)	173.3 (10.5)	172.8 (6.9)	170.3 (9.5)	164.1 (6.1)	173.6 (8.2)	167.4 (6.5)
Sex						
Female	4 (50%)	4 (44%)	5 (63%)	4 (57%)	4 (44%)	7 (88%)
Race						
Asian	1 (13%)	1 (11%)	1 (13%)	1 (14%)	0	0
Black or African American	0	2 (22%)	1 (13%)	2 (29%)	0	1 (13%)
White	7 (88%)	6 (67%)	6 (75%)	4 (57%)	9 (100%)	7 (88%)

Data are mean (SD) or n (%), unless otherwise indicated. Vaccine groups are quantified as per μg of OAg/μg of protein. IM = intramuscular.

**Table 2:** Baseline characteristics in study 2.

	ID				IM		IN			
	0.0059/0.1	0.059/1	0.59/10	Placebo	0.29/5	Placebo	0.29/5	1.2/20	4.8/80	Placebo
n	4	6	6	6	6	1	4	6	6	7
Age (years)	24.8 (5.1)	30.8 (8.3)	26.5 (6.0)	32.0 (8.4)	34.3 (9.2)	30.0	23.5 (5.7)	30.0 (5.9)	27.7 (5.9)	26.9 (6.8)
Weight (kg)	65.88 (9.18)	72.53 (12.10)	71.77 (6.54)	65.53 (12.57)	65.25 (10.47)	56.20	68.70 (8.46)	71.85 (13.56)	64.62 (18.31)	71.20 (11.38)
Height (cm)	165.0 (6.9)	173.8 (10.4)	172.8 (8.7)	165.5 (5.6)	172.8 (12.4)	165.0	172.5 (3.1)	171.8 (8.4)	165.8 (11.7)	170.9 (10.9)
Sex										
Female	4 (100%)	3 (50%)	2 (33%)	4 (67%)	4 (67%)	1 (100%)	3 (75%)	4 (67%)	4 (67%)	5 (71%)
Race										
Asian	1 (25%)	0	0	2 (33%)	0	0	0	0	1 (17%)	0
Black or African American	0	0	0	0	1 (17%)	0	0	2 (33%)	0	1 (14%)
Other	0	0	1 (17%)	0	0	0	0	0	0	0
White	3 (75%)	6 (100%)	5 (83%)	4 (67%)	5 (83%)	1 (100%)	4 (100%)	4 (67%)	5 (83%)	6 (86%)

Data are mean (SD) or n (%), unless otherwise indicated. Vaccine groups are quantified as per μg of OAg/μg of protein. ID = intradermal. IN = intranasal. IM = intramuscular.

### 5.2 Adverse Events

In both studies, vaccines were well tolerated after administration by IM, ID and IN vaccination routes. Solicited events were generally mild to moderate in severity. Vaccine-related unsolicited AEs were uncommon. No subjects experienced SAEs or AEs leading to premature withdrawal from the study and no deaths were reported. Among unsolicited adverse events, 2 cases of grade 3 neutropenia (i.e., ANC 0.5 × 10^9^ to < 1.0 × 10^9^/LL; one case in each study) and 6 cases of grade 2 neutropenia (i.e., ANC 1.0 × 10^9^ to < 1.5 × 10^9^/L; 1 case in study 1 and 5 cases in study 2) were reported. All cases of neutropenia were transient and clinically asymptomatic, and all occurred in vaccines (*N* = 80) and not in placebo recipients (*N* = 22); details of these neutropenia cases have been reported and discussed elsewhere (Muturi-Kioi et al., 2016).

**Fig. 3:**
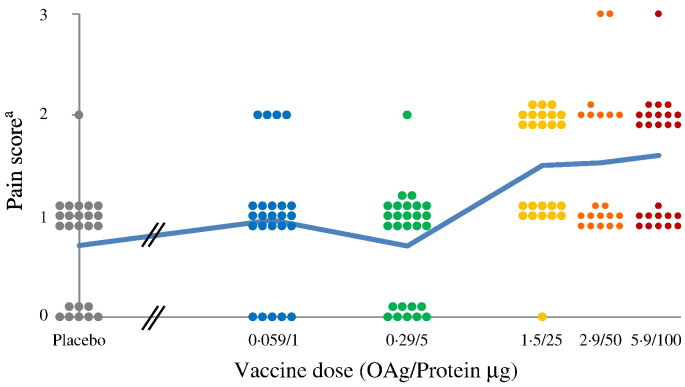
Study 1 - reported maximum local pain following each IM injection. ^a^Pain score 0 = No pain; Pain score 1 = Pain is present, but does not interfere with activity; Pain score 2 = Pain interferes with activity; Pain score 3 = Pain prevents daily activity. Dots represent maximum individual pain reported after each vaccination at different doses. The blue line represents the average pain score as a function of dose. (For interpretation of the references to colour in this figure legend, the reader is referred to the web version of this article.)

In study 1, injection-site pain was the most common solicited local AE and was reported by almost all subjects receiving either vaccine or placebo (Supplementary Table 1). Three subjects (one vaccinated with 2.9/50 μg and two vaccinated with 5.9/100 μg) reported severe local reactions (i.e., pain, erythema and induration) which regressed completely within 24 to 48 h. There was a significant correlation between dose and maximum severity of pain following the first injection (Spearman rank ρ = 0.353, *P* = 0.0118, df = 48) or each of the three injections (Spearman rank ρ = 0.482, *P* = 1.1 × 10^− 9^, df = 141) although subjects receiving 1.5/25, 2.9/50 to 5.9/100 had a similar range of pain scores (Fig. 3). Subjects with pain after the first injection were likely to have pain also after subsequent vaccinations (Spearman rank of maximum pain vaccination 1 vs. maximum pain vaccination 2, ρ = 0.533, *P* = 0.00012, df = 45). Additionally, there was a strong correlation between pain and erythema (Spearman rank ρ = 0.482, *P* = 1.1 × 10–9, df = 141) and pain and induration (Spearman rank ρ = 0.402, *P* = 6.0 × 10–7, df = 142 and Spearman rank ρ = 0.340, *P* = 3.8 × 10–5, df = 139, respectively). Conversely, no significant association was found between pain severity and antibody levels at the time of vaccination. The most commonly reported solicited systemic AEs were myalgia, headache, fatigue and arthralgia (Supplementary Table 1). Only one subject, vaccinated with 0.059/1 μg, had fever, starting on day 5 after the first dose with a peak of 38.3 °C and associated with headache, arthralgia, chills and fatigue. Three subjects reported severe systemic adverse events not accompanied by fever. The first subject, vaccinated with 0.29/5 μg, had severe headache, associated with malaise and fatigue, after the third vaccination. The second subject, vaccinated with 2.9/50 μg, had severe headache, arthralgia, fatigue, malaise and myalgia after the second dose and severe headache, arthralgia, chills, and malaise after the third dose; both second and third doses were also associated with severe local pain. After each vaccination, all reactions resolved by day 5. The third subject, vaccinated with 5.9/100 μg, had severe fatigue after the first vaccination only.

In study 2, the most commonly reported local AEs were injection-site pain following ID and IM vaccination and rhinorrhoea following IN vaccination (Supplementary Table 1); however, none of these local reactions were severe. Most commonly reported solicited systemic AEs in this study were fatigue, headache, malaise and arthralgia (Supplementary Table 1). In two instances, severe systemic adverse events were reported. One subject, vaccinated with 0.29/5 μg dose by the IM route, had severe fatigue starting 5 days post-vaccination and lasting for 1 day. Another subject, vaccinated with 0.29/5 μg vaccine dose by the IN route, developed severe myalgia and fever (peak of 38.5 °C) on the second day post first vaccination. This subject did not have any reaction following the second and third vaccination. One additional subject, vaccinated with 0.29/5 μg dose by the IN route, had fever starting 6 h after vaccination (38.6 °C) and persisting until the third day post vaccination (38.0 °C). All other possibly related unsolicited AEs (except neutropenia reported by one subject receiving 0.12/20 μg dose by IN) were mild to moderate in severity.

### 5.3 Immunogenicity

#### 5.3.1 Pre-existing Antibody

About half of the subjects had measurable anti-LPS antibody pre-vaccination (25/50, study 1; 23/52, Study 2). Of those with measurable antibody, the geometric mean antibody at baseline was higher in study 1 (27.7 Units) than in study 2 (13.4) and this higher GMC reflects a higher number (Lozano et al., 2012) of subjects with baseline titer ≥ 121 in study 1 compared to 1 subject in study 2.

#### 5.3.2 Antibody Response Study 1

**Fig. 4:**
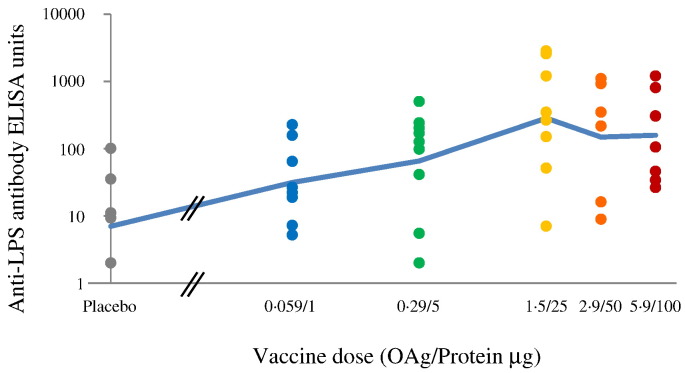
Study 1 - anti-*S. sonnei* LPS antibody response by vaccine dose at day 85. Dots represent individual antibody responses at different doses. The blue line represents the geometric mean ELISA units as a function of dose. (For interpretation of the references to colour in this figure legend, the reader is referred to the web version of this article.)

**Fig. 5:**
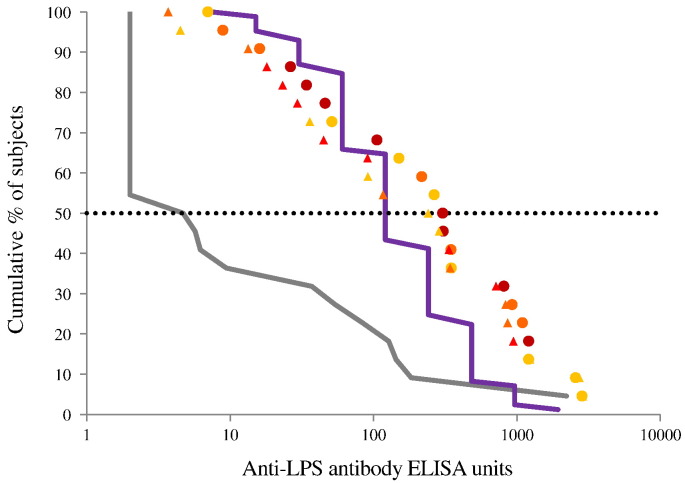
Study 1 - reverse cumulative distribution curves of anti-*S. sonnei* LPS antibody for subjects receiving ≥ 1.5/25 μg by IM route. The grey line is the distribution prior to vaccination. Dots are the distribution on day 85 (i.e., 28 days post 3rd vaccination); triangles are the distribution on day 225 (i.e., 168 days post 3rd vaccination). Subjects received 1.5/25 μg (light orange), 2.9/50 μg (dark orange), or 5.9/100 μg (red). The purple line is the distribution of 87 convalescent subjects. (For interpretation of the references to colour in this figure legend, the reader is referred to the web version of this article.)

There was a significant correlation between dose and antibody response on day 85, i.e. 28 days post 3rd vaccination (Spearman rank ρ = 0.529, *P* = 0.00013, df = 45). The response appears to have peaked with the 1.5/25 μg dose (Fig. 4), and the antibody responses to the 1.5/25, 2.9/50 and 5.9/100 μg doses were not significantly different (Kruskal Wallis test). Therefore these were combined for some post hoc analyses and are collectively called the “high dose group”. Antibody response of subjects receiving 0.29/5 μg IM in study 2 were similar to those of subjects in study 1. As shown in Fig. 5, reverse cumulative antibody distribution at day 85 for the high dose group shows a similar distribution to the antibody responses measured in convalescent sera, with a median of 305 ELISA units compared to 121 for the convalescent patients with a substantial increase in antibody level compared to the pre-vaccination distribution. On the last day of the study, median antibody was still higher than the median in the convalescent patients. (Day 225 median: 241 ELISA units.) Detailed tabulations of the antibody responses calculated according to the protocol analytical plan are shown in the Supplementary data (Supplementary Table 2, Supplementary Table 3, Supplementary Table 4 and Supplementary Figs. 1, 2A, B).

**Fig. 6:**
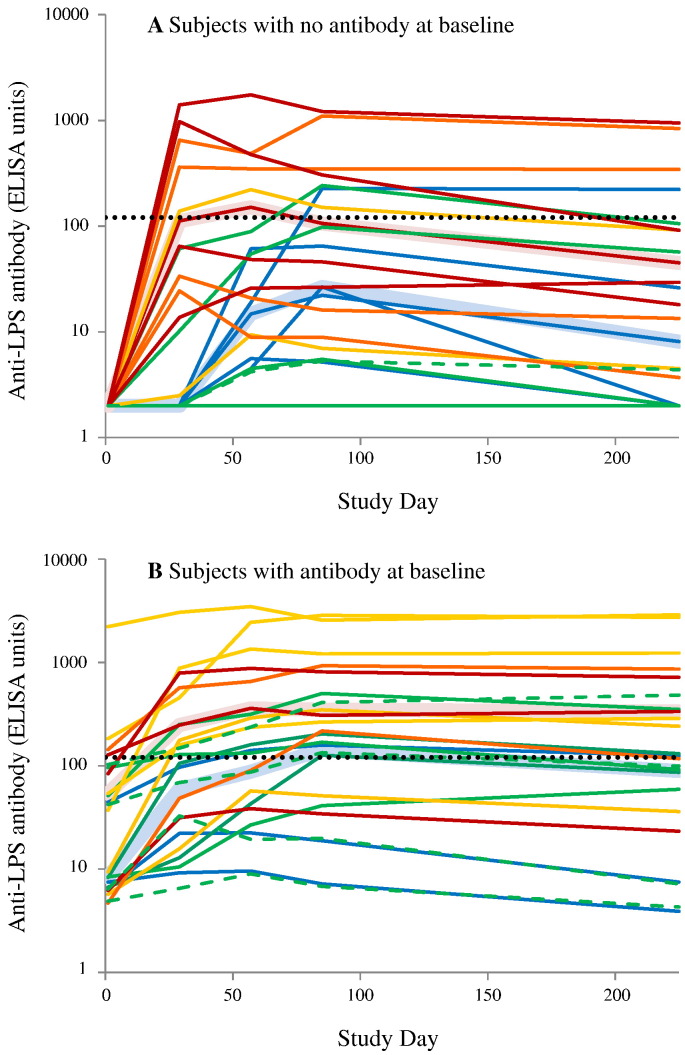
Study 1 and Study 2 - individual antibody responses of subjects receiving vaccines by IM route. Only subjects with antibody levels available on each analysis time point are included. Panel A: Subjects with no detectable antibody at baseline. Subjects with zero detectable antibody were assigned a value of 2 ELISA units (half the detectable limit) to enable plotting. Panel B: subjects with detectable antibody at baseline. Subjects were vaccinated on day 1, 29 and 57 with 0.059/1 μg (blue), 0.29/5 μg (green), 1.5/25 μg (light orange), 2.9/50 μg (dark orange), or 5.9/100 μg (red). Solid line: subjects in Study 1. Dashed green line: subjects in Study 2. Broad pink line: median antibody in the high dose group (subjects receiving ≥ 1.5/25 μg). Broad pale blue line: median antibody in the low dose group (0.059/1 μg and 0.29/5 μg). Dotted line: median antibody (121 ELISA units) measured in convalescent sera. (For interpretation of the references to colour in this figure legend, the reader is referred to the web version of this article.)

Evaluation of the individual antibody responses suggested qualitative and quantitative differences between the response of people with and without pre-existing detectable antibody and to explore these further post hoc analyses were carried out. The individual antibody response of all subjects immunized IM (for both studies) are shown in Fig. 6 for the 44 of 47 subjects for which antibody data was available at all sampling dates. For distinction, antibody responses from the IM group in Study 2 are shown in Fig. 6 using dashed lines.

For subjects with no detectable antibody at baseline (Fig. 6A), the response depended on dose: all volunteers receiving the 2.9/50 μg and 5.9/100 μg, 1 of two receiving 1.5/25 μg, 2 of 4 receiving 0.29/5 μg and none receiving 0.059/1 μg responded after the first vaccination. All but one of the remaining 8 subjects developed detectable antibody after the second vaccination, but two of these (and the non-responder) did not meet the criterion for seroresponse. At the higher doses, most subjects developed maximum antibody after the first vaccination. On average, there was a 47% decrease (median 56%) in antibody levels from day 85 to the last sample at day 225, although this was variable (range: 11% increase to a 100% decrease). Median antibody responses for all high dose subjects peaked at 151 units, thus higher that the reference 121 units in convalescent sera, while peak median antibody in the lower dose groups was 26 units.

For subjects with detectable antibody at baseline (Fig. 6B), all subjects, regardless of dose, had an increase in measured antibody response following the first vaccination, including all of the subjects receiving the two lowest doses. Two subjects in study 1 failed to meet the criterion for seroresponse, one receiving 0.029/5 μg and the person with the highest pre-vaccination antibody (i.e., 2219.6 EU). Unlike the group with no pre-existing antibody, no subject in this group had a peak antibody after the first vaccination. Antibody decayed more slowly from day 85 to day 225 (average 19%, median 30% with a range from a 60% decrease to a 43% increase) than in the group with no detectable antibody at baseline. This difference was marginally significant (Spearman rank ρ = 0.478, *P* = 0.038, df = 17).

#### 5.3.3 Study 2

As indicated above, the antibody responses in the small IM group receiving 0.29/5 μg (Fig. 6, dashed line) were similar to the responses to 0.29/5 μg in the IM group of study 1. However the small group size precludes a more detailed analysis.

##### 5.3.3.1 IN Groups

Of the 17 subjects receiving 0.29/5 μg, 1.2/20 μg or 4.8/80 μg only one subject receiving the lower dose was a seroresponder (8.8 ELISA units to 39.7 after third vaccination) and had any detectable increase in antibody.

##### 5.3.3.2 ID Groups

One of the 4 subjects receiving 0.0059/0.1 μg, just met the seroresponser definition (24.3 ELISA units at baseline, 50.4 ELISA units after the first vaccination). None of the other 5 volunteers in this group, the 6 in the 0.059/1 μg, or the 4 analysable subjects who received 0.59/10 μg had a detectable antibody increase. Two subjects in the higher dose ID group could not be analysed. One had relatively high, stable antibody levels (157 and 190 ELISA units after the first and second vaccination, respectively), but we were unable to record the baseline level). The second withdrew after the first vaccination. Total, secretory and *S. sonnei* specific IgA were evaluated in stool samples from cohort 3 of study 2. No significant correlation was identified between vaccination (by any route) and IgA/sIgA increase (data not shown).

Cell mediated immunity was only planned and analysed in subjects from cohort 3 of study 2; MBC did not show significant differences in the number of subjects with detectable frequencies of IgA and IgG MBC specific for the OAg vaccine antigen, its carrier GMMA and the negative control Human Serum Albumin (HSA). Same was true for plasmablasts, analysed at day 7 and showing, overall, low frequencies of vaccine antigen specific plasmablasts, both against the OAg and the GMMA carrier (data not shown).

As CMI studies had been planned only for a subset of subjects immunized in study 2, the cellular response of the vaccine formulations administered intramuscularly in study 1 and associated with the highest serological response could not be established. Complete faecal IgA studies (including results from study 1, still not tested) will be reported elsewhere.

## 6 Discussion

For any new technology a primary concern is safety and reactogenicity, especially for a vaccine that potentially contains powerful stimulators of the innate immune system. In these studies, we saw a correlation between dose and local reactions, but even at the highest dose of 5.9/100 μg the vaccine had an acceptable reactogenicity profile, which was less than that reported for the licensed, Outer Membrane Vesicles (OMV) containing vaccine, 4CMenB (Toneatto et al., 2011). Importantly, as 1790GAHB comprises the outer membrane of *Shigella*, it contains appreciable levels of lipid A, which in the 1790-GMMA producing strain is genetically modified to be penta-acylated (Gerke et al., 2015) (320 nmoles of lipid A per 100 μg of protein, data not shown) and other potential activators of innate immune responses (e.g. lipidated proteins). In pre-clinical studies, rabbits receiving 5.9/100 μg IM had an average temperature increase of 0.4 °C compared to controls at 4 h after injection (Gerke et al., 2015). In the clinical studies we saw no consistent temperature change as a function of dose and no significant pyrogenic response over the 4 hour post vaccination. As the ratio of lipid A to protein is relatively constant and the structure of the outer membrane is similar in GMMA from related organisms (e.g. from other *Shigella* species), doses of up to at least 100 μg of protein are likely to be acceptable even though the OAg content may vary considerably, depending on the average size of the OAg in each GMMA.

These studies compared three routes of immunization (IN, ID and IM), which have different theoretical advantages: IN provides a needle free vaccination route and may elicit stronger mucosal immunity (Zaman et al., 2013), ID may provide dose sparing (Zehrung et al., 2013) and IM may provide strong systemic immunity and, as the standard vaccination route, may be easier to introduce into routine public health vaccination programs. All three routes gave strong IgG responses in rabbits when tested at the maximum human dose (Gerke et al., 2015), with the ID route giving significantly higher antibody responses than IM, and the IN route lower peak responses than the IM. These preclinical data contrast with the clinical results, which show no detectable immune responses in most human subjects vaccinated via the ID or IN routes, even at the maximum doses tested. Therefore, data suggest that ID vaccination is unlikely to be a useful route for GMMA adsorbed on aluminium hydroxide.

Alternatively, IN results seem to be consistent with human IN vaccination studies of *S. flexneri* 2a LPS adsorbed onto detergent extracted *Neisseria meningitidis* OMV where no or minimal circulating antibody was detected after two doses of 90 μg of LPS, but significant IgG and serum IgA was measured following 360 μg of LPS (Fries et al., 2001). While these subjects were given a different formulation, and a different serotype of *Shigella*, measured in a different way (μg of total LPS vs μg of OAg), never-the-less, these data suggest that the maximum IN dose in our study of 4.8/80 μg is too low to definitively rule out the use of IN route with substantially larger doses. They also suggest that immunogenicity responses in rabbits are not predictive of human results.

There is an interest in *Shigella* vaccines that induce strong mucosal immunity (i.e. sIgA responses) (Mani et al., 2016). Our limited results, from subjects immunized with 1790GAHB in cohort 3 of trial 2, did not show any significant IgA response at the tested doses (i.e., 0.59/10 μg ID, 4.8/80 μg IN and 0.29/5 μg IM). We acknowledge that these results do not preclude the possibility that the use of an adjuvant, like double mutant LT, might induce a more robust mucosal response using the ID or IN routes. However, the key pathogenic step in shigellosis is the invasion of gut epithelial cells from the basal surface by bacteria that have crossed into the systemic circulation via M cells (Zychlinsky et al., 1994). Thus, although sIgA may play a role in reducing the bacterial inoculum, IgG is likely to play a key role in preventing disease. This central role for systemic IgG is consistent with the demonstrated field efficacy of an IM conjugate vaccine (Cohen et al., 1997) and the correlation between natural induced anti-OAg IgG levels and subsequent risk of infection (Cohen et al., 1991). Patients recovering from infection with *Shigella* have strain specific immunity against subsequent infection (Cohen et al., 1989). As strain specificity is determined by the anti-OAg response, we postulate that magnitude of anti-IgG OAg responses in convalescent patients is an indicator of the relevance of the IgG responses elicited in the studies with 1790GAHB. Importantly, in the study 1, subjects receiving ≥ 1.5/25 μg dose IM had a reverse cumulative antibody distribution similar to that of convalescent patients, but with a higher median antibody level (305 vs 121 ELISA-units) and a relatively slow decay in antibody responses (median level of 241 at day 225, still exceeding the median level in convalescent patients).

Approximately half of the subjects in these studies had detectable anti-*S. sonnei* LPS antibody prior to vaccination. What induced these antibodies is not known: e.g., asymptomatic or undiagnosed prior infection with *S. sonnei* or exposure to organisms with a related O antigen e.g. the Gram-negative *Plesiomonas shigelloides* serotype O17, which may cause mild waterborne gastroenteritis in humans (Kubler-Kielb et al., 2008). As subjects with pre-existing antibody tended to have stronger responses to the vaccine and different pattern of boosting and decay kinetics, it will be important to consider the impact of pre-exposure in future trials by evaluating the response to different levels of pre-existing antibody in populations that may benefit most from a *Shigella* vaccine.

These data support the development of a multivalent GMMA Shigella vaccine. More importantly, the peak antibody response occurred at a dose of 1.5/25 μg and the maximum dose tested of 5.9/100 μg was well tolerated. These results not only support development of a multivalent *Shigella* vaccine, but also other vaccines, built around the affordable GMMA technology, with the potential to impact on major global diseases of developing countries.

The following are the supplementary data related to this article.

Supplementary Fig. 1 Median anti-*S. sonnei* LPS IgG (EU/mL), at baseline, 1 month after first, second and third vaccination and at 6 months after third vaccination – modified FAS vaccine group (all subjects, all groups). Supplementary Fig. 1

Supplementary Fig. 2 A: median anti-*S. sonnei* LPS IgG (EU/mL), at baseline, 1 month after first, second and third vaccination and at 6 months after third vaccination, in subjects with detectable antibodies at baseline (subjects with data at all visits). B: median anti-*S. sonnei* LPS IgG (EU/mL), at baseline, 1 month after first, second and third vaccination and at 6 months after third vaccination, in subjects with not-detectable antibodies at baseline (subjects with data at all visits). Supplementary Fig. 2

Supplementary Fig. 6D Study 1 - individual antibody responses of subjects vaccinated on day 1, 29 and 57 with 1.5/25 μg (light orange), 2.9/50 μg (dark orange), or 5.9/100 μg (red). Subjects with no detectable antibody at baseline are indicated with solid lines and subjects with detectable antibody at baseline with dashed lines. (Subjects with data at all visits.) (For interpretation of the references to colour in this figure legend, the reader is referred to the web version of this article.) Supplementary Fig. 6D

Supplementary Table 1 Number of subjects with solicited local and systemic adverse events reported from 30 min through 7 days after any injection. Supplementary Table 1

Supplementary Table 2 Trial 1 – ELISA GMCs, GMRs (95% CIs) and median at baseline, 1 month after first, second and third vaccination and at 6 months after third vaccination. Supplementary Table 2

Supplementary Table 3 Percentage (95% CI) of subjects with anti-LPS antibody titer > 121 EU after vaccination*. Supplementary Table 3

Supplementary Table 4 Trial 2 – ELISA GMCs, GMRs (95% CIs) and median at baseline, 1 month after first, second and third vaccination and at 6 months after third vaccination. Supplementary Table 4
