# Supplementary figures and images for: Safety Profile and Immunologic Responses of a Novel Vaccine Against Shigella sonnei Administered Intramuscularly, Intradermally and Intranasally: Results From Two Parallel Randomized Phase 1 Clinical Studies in Healthy Adult Volunteers in Europe
Source: eBioMedicine. 2017 Jul 15;22:164–72. doi: 10.1016/j.ebiom.2017.07.013 (PMC5552227; doi:10.1016/j.ebiom.2017.07.013)

**Figure 1**

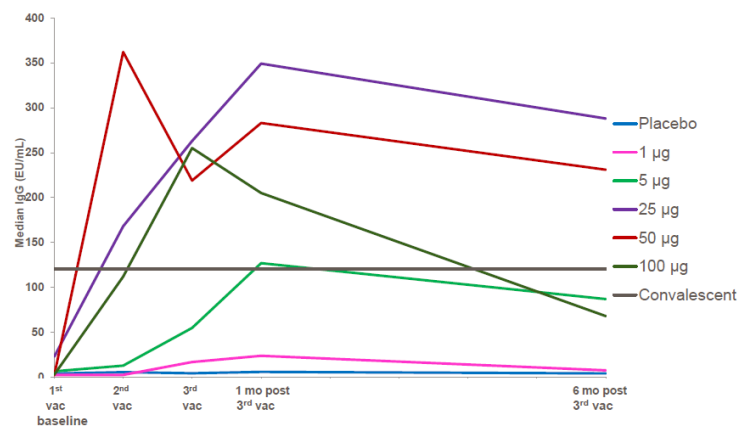

Supplement: Supplementary Fig. 1 — Median anti-S. sonnei LPS IgG (EU/mL), at baseline, 1 month after first, second and third vaccination and at 6 months after third vaccination – modified FAS vaccine group (all subjects, all groups). [file mmc1.pdf]

Figure 2a

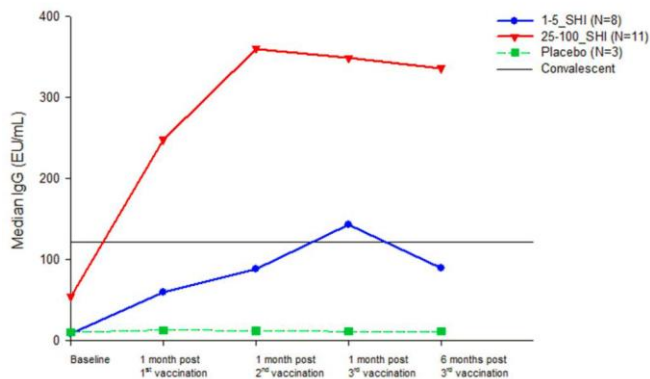

Figure 2b

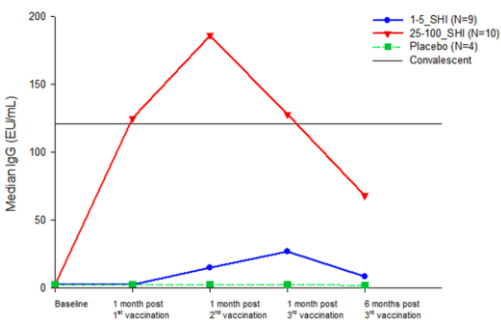

Supplement: Supplementary Fig. 2 — A: median anti-S. sonnei LPS IgG (EU/mL), at baseline, 1 month after first, second and third vaccination and at 6 months after third vaccination, in subjects with detectable antibodies at baseline (subjects with data at all visits). B: median anti-S. sonnei LPS IgG (EU/mL), at baseline, 1 month after first, second and third vaccination and at 6 months after third vaccination, in subjects with not-detectable antibodies at baseline (subjects with data at all visits). [file mmc2.pdf]

**Figure 6D**

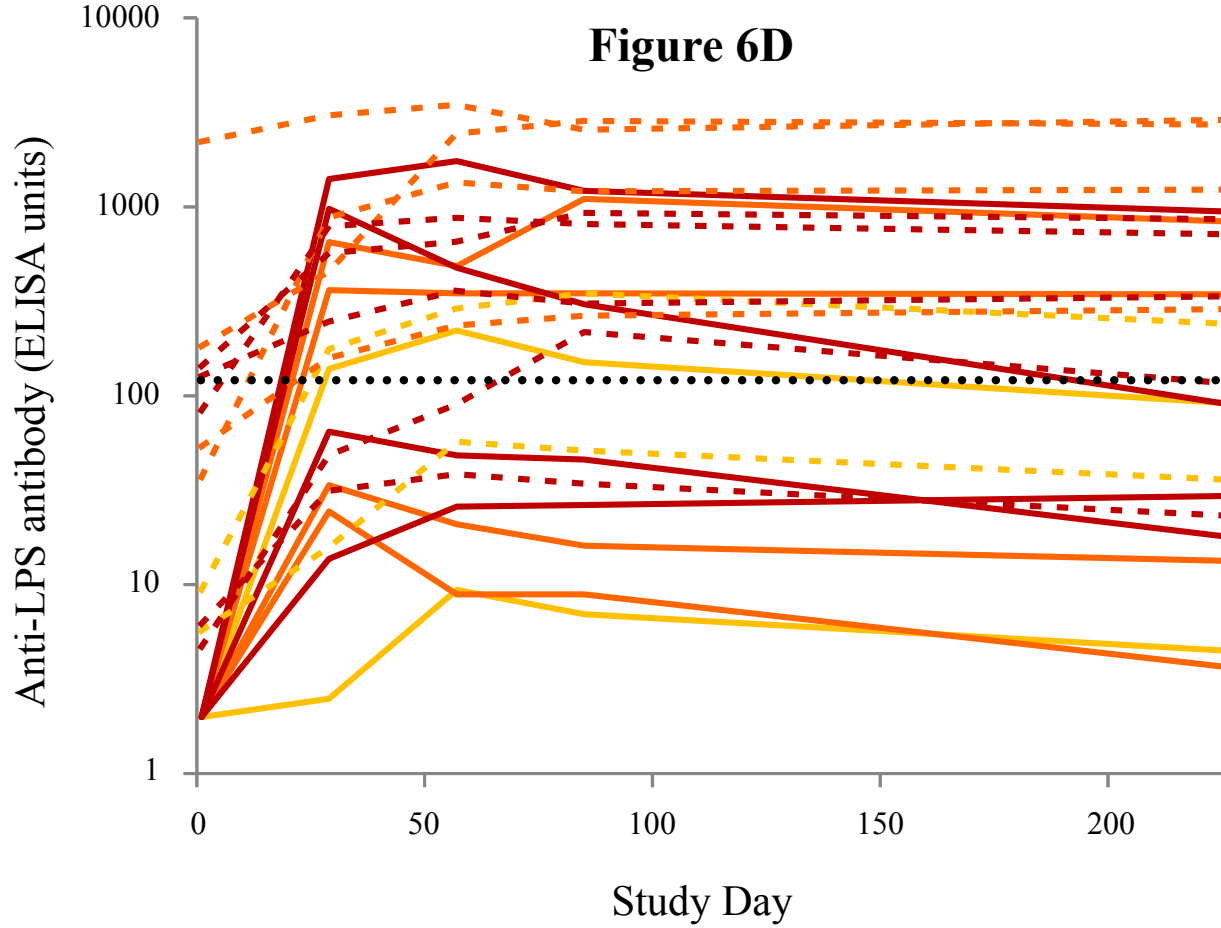

Supplement: Supplementary Fig. 6D — Study 1 - individual antibody responses of subjects vaccinated on day 1, 29 and 57 with 1.5/25 μg (light orange), 2.9/50 μg (dark orange), or 5.9/100 μg (red). Subjects with no detectable antibody at baseline are indicated with solid lines and subjects with detectable antibody at baseline with dashed lines. (Subjects with data at all visits.) (For interpretation of the references to colour in this figure legend, the reader is referred to the web version of this article.) [file mmc3.pdf]
