# Supplementary material for: Safety Profile and Immunologic Responses of a Novel Vaccine Against Shigella sonnei Administered Intramuscularly, Intradermally and Intranasally: Results From Two Parallel Randomized Phase 1 Clinical Studies in Healthy Adult Volunteers in Europe
Source: eBioMedicine. 2017 Jul 15;22:164–72. doi: 10.1016/j.ebiom.2017.07.013 (PMC5552227; doi:10.1016/j.ebiom.2017.07.013)
Supplement: Supplementary Table 1 — Number of subjects with solicited local and systemic adverse events reported from 30 min through 7 days after any injection. [file mmc4.pdf]

Supplementary Table 1: Number of subjects with solicited local and systemic adverse events reported from 30 minutes through 7 days after any injection

Study		Trial 1 - H03_01TP						Trial 2 - H03_02TP									
Vaccine group		1790GAHB					Placebo	1790GAHB				Placebo		1790GAHB			Placebo
		IM					IM	ID			IM	ID	IM	IN			IN
		0·059/1	0·29/5	1·5/25	2·9/50	5·9/100		0·0059/0·1	0·059/1	0·59/10	0·29/5			0·29/5	1·2/20	4·8/80	
		N=8	N=9	N=8	N=8	N=9	N=8	N=4	N=6	N=6	N=6	N=6	N=1	N=4	N=6	N=6	N=7
**Local reactions, n**	**Severity**																
Pain at injection site	Any[a]	8	9	8	8	9	6	0	2	3	5	2	1	NA	NA	NA	NA
	Severe	0	0	0	1	1	0	0	0	0	0	0	0	NA	NA	NA	NA
Erythema	Any[b]	0	0	0	0	3	1	0	0	1	0	0	0	NA	NA	NA	NA
	Severe	0	0	0	0	1	0	0	0	0	0	0	0	NA	NA	NA	NA
Induration	Any[b]	0	0	0	2	2	0	0	0	0	0	0	0	NA	NA	NA	NA
	Severe	0	0	0	1	0	0	0	0	0	0	0	0	NA	NA	NA	NA
Swelling	Any[b]	NA	NA	NA	NA	NA	NA	0	0	0	NA	0	NA	NA	NA	NA	NA
	Severe	NA	NA	NA	NA	NA	NA	0	0	0	NA	0	NA	NA	NA	NA	NA
Facial edema	Any[a]	NA	NA	NA	NA	NA	NA	NA	NA	NA	NA	NA	NA	0	0	1	0
	Severe	NA	NA	NA	NA	NA	NA	NA	NA	NA	NA	NA	NA	0	0	0	0
Nasal pain	Any[a]	NA	NA	NA	NA	NA	NA	NA	NA	NA	NA	NA	NA	1	1	1	0
	Severe	NA	NA	NA	NA	NA	NA	NA	NA	NA	NA	NA	NA	0	0	0	0
Rhinorrhea	Any[a]	NA	NA	NA	NA	NA	NA	NA	NA	NA	NA	NA	NA	2	4	4	1
	Severe	NA	NA	NA	NA	NA	NA	NA	NA	NA	NA	NA	NA	0	0	0	0
**Systemic reactions, n**	**Severity[c]**																
Headache	Any	3	3	2	4	2	3	1	3	1	1	3	1	4	3	3	4
	Severe	0	1	0	1	0	0	0	0	0	0	0	0	0	0	0	0
Arthralgia	Any	2	0	2	3	3	1	1	3	0	0	1	0	2	1	1	0
	Severe	0	0	0	1	0	0	0	0	0	0	0	0	0	0	0	0
Chills	Any	2	0	1	1	0	1	0	1	0	0	1	0	2	2	1	0
	Severe	0	0	0	1	0	0	0	0	0	0	0	0	0	0	0	0
Fatigue	Any	4	3	6	5	5	5	2	5	0	2	3	1	3	3	4	4
	Severe	0	0	0	1	1	0	0	0	0	1	0	0	0	0	0	0
Malaise	Any	0	1	0	1	2	0	1	2	1	1	1	0	2	1	4	2
	Severe	0	0	0	1	0	0	0	0	0	0	0	0	0	0	0	0
Myalgia	Any	5	1	3	4	5	3	1	2	1	1	0	1	2	1	2	1
	Severe	0	0	0	1	0	0	0	0	0	0	0	0	1	0	0	0
Fever	≥ 38 °C	1	0	0	0	0	0	0	0	0	0	0	0	2	0	0	0

≥40°C	0	0	0	0	0	0	0	0	0	0	0	0	0	0	0	0
**Other, n**																
Prevention of pain or fever	0	0	0	0	0	0	0	0	0	0	0	0	0	0	0	0
Treatment of pain or fever	2	2	2	1	0	1	1	1	1	1	0	1	1	1	1	0

IM=intramuscular. ID=intradermal. IN=intranasal. NA=not applicable

a Grading for injection site pain/nasal pain/facial edema/rhinorrhea was: grade 0 (absent) no pain; grade 1 (mild) present but does not interfere with activity; grade 2 (moderate) interferes with activity; grade 3 (severe) prevents daily activity. Any refers to injection site pain/nasal pain/facial edema/rhinorrhea at least grade 1

b Any refers to erythema/induration/swelling ≥25 mm in diameter, including severe erythema/induration/swelling (>100 mm).

c Grading for headache, arthralgia, chills, fatigue, malaise, myalgia was: grade 0 (absent) no reaction; grade 1 (mild) present but does not interfere with activity; grade 2 (moderate) interferes with activity; grade 3 (severe) prevents daily activity
