# Supplementary material for: Safety Profile and Immunologic Responses of a Novel Vaccine Against Shigella sonnei Administered Intramuscularly, Intradermally and Intranasally: Results From Two Parallel Randomized Phase 1 Clinical Studies in Healthy Adult Volunteers in Europe
Source: eBioMedicine. 2017 Jul 15;22:164–72. doi: 10.1016/j.ebiom.2017.07.013 (PMC5552227; doi:10.1016/j.ebiom.2017.07.013)
Supplement: Supplementary Table 2 — Trial 1 – ELISA GMCs, GMRs (95% CIs) and median at baseline, 1 month after first, second and third vaccination and at 6 months after third vaccination. [file mmc5.pdf]

Supplementary Table 2: Trial 1 – ELISA GMCs, GMRs (95% CIs) and median at baseline, 1 month after first, second and third vaccination and at 6 months after third vaccination

Vaccine Group	*S. sonnei* (1790GAHB) - IM					Placebo - IM
	**0·059/1** N=8	**0·29/5** N=9	**1·5/25** N=8	**2·9/50** N=7	**5·9/100** N=9	**Placebo** N=8
Baseline						
GMC (95% CI)	4·71 (2·02-11)	7·74 (2·72-22)	27 (3·75-191)	8·78 (1·2-64)	6·91 (2·03-24)	6·37 (2·38-17)
Median	2·57	6·7	23	2·62	2·92	4·23
n	8	9	8	7	9	8
1 month after first vac.						
GMC (95% CI)	6·2 (1·97-20)	23 (6·13-86)	141 (21-962)	186 (40-855)	157 (45-546)	6·35 (2·61-15)
Median	2·57	13	168	362	112	5·76
n	8	9	8	7	9	8
1 month after first vac./Baseline						
GMR (95% CI)	1·32 (0·89-1·95)	2·96 (1·21-7·27)	5·25 (1·48-19)	21 (4·58-98)	23 (4·87-106)	1 (0·78-1·27)
Median	1	1·93	2·85	12	11	1
n	8	9	8	7	9	8
1 month after second vac.						
GMC (95% CI)	18 (6·95-49)	41 (11-148)	314 (60-1646)	111 (17-717)	193 (52-707)	6·56 (2·5-17)
Median	17	55	263	219	255	4·56
n	8	9	8	6	8	8
1 month after second vac./Baseline						
GMR (95% CI)	3·91 (1·79-8·54)	5·32 (1·92-15)	12 (3·79-36)	22 (3·72-134)	28 (5·62-136)	1·03 (0·93-1·14)
Median	3·31	6·14	12	15	16	0·98
n	8	9	8	6	8	8
1 month after third vac.						
GMC (95% CI)	32 (10-98)	67 (17-269)	486 (120-1959)	149 (17-1289)	159 (47-539)	7·71 (2·31-26)
Median	24	127	349	283	205	6·11
n	8	9	7	6	8	8
1 month after third vac./Baseline						
GMR (95% CI)	6·69 (1·97-23)	8·64 (2·59-29)	13 (3·62-46)	30 (3·97-227)	23 (5·17-101)	1·21 (0·79-1·87)
Median	6·11	9·76	16	27	15	0·95
n	8	9	7	6	8	8
6 Months after third vac.						
GMC (95% CI)	12 (2·66-57)	42 (9·96-178)	418 (87-2002)	106 (9·89-1143)	102 (27-383)	5·05 (1·65-15)
Median	7·8	87	288	231	68	4·4
n	8	9	7	6	8	7
6 Months after third vac./Baseline						
GMR (95% CI)	2·62 (0·62-11)	5·43 (1·63-18)	11 (3·6-34)	21 (2·48-185)	15 (3·86-55)	0·98 (0·7-1·38)
Median	1·98	7·06	15	16	9·33	1·04
n	8	9	7	6	8	7

CI =confidence interval. GMR = geometric mean ratio. GMC = geometric mean concentration. vac.= vaccination.
