# Supplementary material for: Safety Profile and Immunologic Responses of a Novel Vaccine Against Shigella sonnei Administered Intramuscularly, Intradermally and Intranasally: Results From Two Parallel Randomized Phase 1 Clinical Studies in Healthy Adult Volunteers in Europe
Source: eBioMedicine. 2017 Jul 15;22:164–72. doi: 10.1016/j.ebiom.2017.07.013 (PMC5552227; doi:10.1016/j.ebiom.2017.07.013)
Supplement: Supplementary Table 3 — Percentage (95% CI) of subjects with anti-LPS antibody titer > 121 EU after vaccination*. [file mmc6.pdf]

Supplementary Table 3: Percentage (95% CI) of subjects with anti-LPS antibody titer >121 EU after vaccination*

Trial	Vaccine group		28 days after 1st vaccination	28 days after 2nd vaccination	28 days after 3rd vaccination	6 months after last vaccination
1	0·059/1	%	0%	13%	25%	25%
	IM	95% CI	(0-37)	(0-53)	(3-65)	(3-65)
		n	8	8	8	8
	0·29/5	%	22%	33%	56%	22%
	IM	95% CI	(3-60)	(7-70)	(21-86)	(3-60)
		n	9	9	9	9
	1·5/25	%	75%	75%	86%	71%
	IM	95% CI	(35-97)	(35-97)	(42-100)	(29-96)
		n	8	8	7	7
	2·9/50	%	57%	50%	67%	50%
	IM	95% CI	(18-90)	(12-88)	(22-96)	(12-88)
		n	7	6	6	6
	5·9/100	%	44%	63%	50%	38%
	IM	95% CI	(14-79)	(24-91)	(16-84)	(9-76)
		n	9	8	8	8
	Placebo	%	0%	0%	0%	0%
	IM	95% CI	(0-37)	(0-37)	(0-37)	(0-41)
		n	8	8	8	7
2	0·0059/0·1	%	0%	0%	0%	0%
	ID	95% CI	(0-60)	(0-60)	(0-60)	(0-60)
		n	4	4	4	4
	0·059/1	%	0%	0%	0%	0%
	ID	95% CI	(0-46)	(0-52)	(0-46)	(0-46)
		n	6	5	6	6
	0·59/10	%	20%	20%	20%	0%
	ID	95% CI	(1-72)	(1-72)	(1-72)	(0-52)
		n	5	5	5	5
	0·29/5	%	0%	0%	0%	0%
	IN	95% CI	(0-60)	(0-60)	(0-60)	(0-60)
		n	4	4	4	4
	1·2/20	%	17%	17%	17%	17%
	IN	95% CI	(0-64)	(0-64)	(0-64)	(0-64)
		n	6	6	6	6
	4·8/80	%	0%	0%	0%	0%
	IN	95% CI	(0-46)	(0-52)	(0-46)	(0-46)
		n	6	5	6	6
	0·29/5	%	17%	17%	33%	17%
	IM	95% CI	(0-64)	(0-64)	(4-78)	(0-64)
		n	6	6	6	6
	Placebo	%	0%	0%	0%	0%
		95% CI	(0-23)	(0-23)	(0-25)	(0-26)
		n	14	14	13	12

CI =confidence interval. Vaccine groups are quantified as per µg of OAg/µg of protein. ID= intradermal. IN= intranasal. IM=intramuscular.
