# Supplementary material for: Safety Profile and Immunologic Responses of a Novel Vaccine Against Shigella sonnei Administered Intramuscularly, Intradermally and Intranasally: Results From Two Parallel Randomized Phase 1 Clinical Studies in Healthy Adult Volunteers in Europe
Source: eBioMedicine. 2017 Jul 15;22:164–72. doi: 10.1016/j.ebiom.2017.07.013 (PMC5552227; doi:10.1016/j.ebiom.2017.07.013)
Supplement: Supplementary Table 4 — Trial 2 – ELISA GMCs, GMRs (95% CIs) and median at baseline, 1 month after first, second and third vaccination and at 6 months after third vaccination. [file mmc7.pdf]

**Supplementary Table 4: Trial 2 – ELISA GMCs, GMRs (95% CIs) and median at baseline, 1 month after first, second and third vaccination and at 6 months after third vaccination**

| | | ID | | | IN | | IM | |
|---|---|---|---|---|---|---|---|---|
| **Vaccine Group** | **0·0059/0·1** | **0·059/1** | **0·59/10** | **0·29/5** | **1·2/20** | **4·8/80** | **0·29/5** | **Placebo** |
| | N=4 | N=6 | N=5 | N=4 | N=6 | N=6 | N=6 | N=14 |
| Baseline | | | | | | | | |
| GMC (95% CI) | 7·71 | 1·8 | 5·14 | 3·65 | 14 | 2·11 | 8·27 | 3·29 |
| | (0·55-107) | (1·66-1·95) | (2·18-12) | (0·61-22) | (0·97-205) | (1·1-4·03) | (1·34-51) | (1·93-5·6) |
| Median | 13 | 1·78 | 5·8 | 2·63 | 6·35 | 1·68 | 5·65 | 1·86 |
| | 4 | 6 | 5 | 4 | 6 | 6 | 6 | 14 |
| n | | | | | | | | |
| 1 month after first vac. | | | | | | | | |
| GMC (95% CI) | 9·4 | 2·11 | 12 | 4·08 | 13 | 2·09 | 13 | 3·58 |
| | (0·48-184) | (1·43-3·11) | (1·58-96) | (0·76-22) | (0·95-187) | (1·03-4·23) | (1·69-102) | (2·1-6·08) |
| Median | 23 | 1·86 | 9·5 | 3·23 | 5·35 | 1·55 | 20 | 3·01 |
| n | 4 | 6 | 5 | 4 | 6 | 6 | 6 | 14 |
| 1 month after first vac./Baseline | | | | | | | | |
| GMR (95% CI) | 1·22 | 1·17 | 1·54 | 1·12 | 0·95 | 0·99 | 1·59 | 1·09 |
| | (0·69-2·15) | (0·79-1·73) | (0·61-3·9) | (0·86-1·45) | (0·76-1·17) | (0·9-1·09) | (0·84-3) | (0·76-1·54) |
| Median | 1·03 | 1·1 | 1·24 | 1·11 | 0·95 | 1 | 1·38 | 1·05 |
| n | 4 | 6 | 4 | 4 | 6 | 6 | 6 | 14 |
| 1 month after second vac. | | | | | | | | |
| GMC (95% CI) | 9·09 | 2·32 | 12 | 3·78 | 15 | 1·72 | 17 | 3·28 |
| | (0·49-167) | (1·2-4·5) | (1·45-107) | (0·61-23) | (1·04-219) | (1·59-1·86) | (2·47-120) | (1·98-5·42) |
| Median | 23 | 2·03 | 7·5 | 2·7 | 7·55 | 1·75 | 14 | 1·91 |
| n | 4 | 5 | 5 | 4 | 6 | 5 | 6 | 14 |
| 1 month after second vac./Baseline | | | | | | | | |
| GMR (95% CI) | 1·18 | 1·32 | 1·49 | 1·03 | 1·07 | 1·05 | 2·09 | 1 |
| | (0·72-1·93) | (0·66-2·62) | (0·6-3·7) | (0·9-1·19) | (0·96-1·19) | (0·98-1·13) | (1·45-3) | (0·89-1·11) |
| Median | 1·02 | 1·09 | 1·15 | 1·07 | 1·05 | 1·04 | 2·18 | 1·03 |
| n | 4 | 5 | 4 | 4 | 6 | 5 | 6 | 14 |
| 1 month after third vac. | | | | | | | | |
| GMC (95% CI) | 8·63 | 1·75 | 14 | 3·85 | 19 | 2·21 | 20 | 3·49 |
| | (0·43-173) | (1·62-1·89) | (2·43-85) | (0·79-19) | (1·31-270) | (1·23-3·97) | (2·29-179) | (1·98-6·14) |
| Median | 20 | 1·68 | 9·9 | 3·11 | 23 | 1·75 | 13 | 2·03 |
| n | 4 | 6 | 5 | 4 | 6 | 6 | 6 | 13 |
| 1 month after third vac./Baseline | | | | | | | | |
| GMR (95% CI) | 1·12 | 0·97 | 1·87 | 1·05 | 1·34 | 1·05 | 2·45 | 1·11 |
| | (0·59-2·12) | (0·93-1·01) | (0·82-4·25) | (0·73-1·51) | (0·7-2·56) | (0·97-1·14) | (1·41-4·25) | (0·93-1·32) |
| Median | 0·92 | 0·99 | 1·93 | 1·07 | 0·99 | 1·04 | 3·14 | 1·07 |
| n | 4 | 6 | 4 | 4 | 6 | 6 | 6 | 13 |
| 6 Months after third vac. | | | | | | | | |
| GMC (95% CI) | 6·23 | 1·7 | 6·69 | 3·2 | 16 | 2·58 | 15 | 2·59 |
| | (0·55-70) | (1·7-1·7) | (0·91-49) | (0·78-13) | (1·05-254) | (1·36-4·9) | (1·56-148) | (1·62-4·13) |
| Median | 8·95 | 1·7 | 6·2 | 2·65 | 10 | 1·87 | 5·8 | 1·77 |
| n | 4 | 6 | 5 | 4 | 6 | 6 | 6 | 12 |
| 6 Months after third vac./Baseline | | | | | | | | |
| GMR (95% CI) | 0·81 | 0·95 | 0·83 | 0·88 | 1·16 | 1·22 | 1·84 | 0·77 |
| | (0·35-1·85) | (0·87-1·02) | (0·27-2·56) | (0·56-1·36) | (0·85-1·58) | (0·91-1·64) | (0·93-3·63) | (0·55-1·08) |
| Median | 0·91 | 0·96 | 0·97 | 0·98 | 1·04 | 1·11 | 1·79 | 0·97 |
| n | 4 | 6 | 4 | 4 | 6 | 6 | 6 | 12 |

CI =confidence interval. Vaccine groups are quantified as per µg of OAg/µg of protein. ID= intradermal. IN= intranasal. IM=intramuscular. GMR = geometric mean ratio. GMC = geometric mean concentration. vac.= vaccination.
